# Modelling the Performance of Electrically Conductive Nanofiltration Membranes

**DOI:** 10.3390/membranes13060596

**Published:** 2023-06-12

**Authors:** Alexey A. Kapitonov, Ilya I. Ryzhkov

**Affiliations:** 1 Institute of Computational Modelling SB RAS, Akademgorodok 50-44, 660036 Krasnoyarsk, Russia; 2 School of Space and Information Technology, Siberian Federal University, Svobodny 79, 660041 Krasnoyarsk, Russia

**Keywords:** nanofiltration, electrically conductive membrane, ionic selectivity, mathematical modelling

## Abstract

Electrically conductive membranes are a class of stimuli-responsive materials, which allow the adjustment of selectivity for and the rejection of charged species by varying the surface potential. The electrical assistance provides a powerful tool for overcoming the selectivity–permeability trade-off due to its interaction with charged solutes, allowing the passage of neutral solvent molecules. In this work, a mathematical model for the nanofiltration of binary aqueous electrolytes by an electrically conductive membrane is proposed. The model takes into account the steric as well as Donnan exclusion of charged species due to the simultaneous presence of chemical and electronic surface charges. It is shown that the rejection reaches its minimum at the potential of zero charge (PZC), where the electronic and chemical charges compensate for each other. The rejection increases when the surface potential varies in positive and negative directions with respect to the PZC. The proposed model is successfully applied to a description of experimental data on the rejection of salts and anionic dyes by PANi–PSS/CNT and MXene/CNT nanofiltration membranes. The results provide new insights into the selectivity mechanisms of conductive membranes and can be employed to describe electrically enhanced nanofiltration processes.

## 1. Introduction

Nowadays, the most common membrane technologies for the separation, purification, and concentration of solutions are baromembrane processes, which include microfiltration (MF), ultrafiltration (UF), nanofiltration (NF), and reverse osmosis (RO) [1]. Nanofiltration is used to remove hardness salts, heavy metal salts, and low-molecular-weight organic compounds [2]. Membranes for nanofiltration have pore sizes in the range of 0.5–2 nm and can remove species with a molecular weight of 100–2000 Dalton at a transmembrane pressure of 1–20 atm. In comparison with reverse osmosis, which has been widely employed for desalination of water, nanofiltration is characterized by higher flux, lower pressure, and consequently, lower energy consumption. Nanofiltration is effective at removing multivalent salt ions but shows relatively low rejection (around 50–70%) of monovalent salt ions [3].

The main directions of NF membrane development are increasing retention and selectivity, enhancing performance, reducing fouling, and increasing chemical stability and service life [4]. High selectivity can be achieved in NF membranes due to their sieving separation mechanism, which allows the removal of both ionic solutes and neutral molecules. Reducing the pore size increases the selectivity of the membrane but decreases its permeability.

The separation of electrolyte solutions is realized through the sieving effect and the Donnan exclusion mechanism [5]. The presence of a fixed charge in the selective membrane layer causes a decrease in the concentration of ions with the same charge sign inside the pores and leads to an increase in selectivity. By varying the charge, the selectivity of a membrane can be significantly improved without compromising its permeability. This approach can be realized in membranes with electrically conductive surfaces, the charge of which can be controlled by varying the surface potential [6]. This gives an additional degree of freedom in terms of adjusting the selective properties of the membrane relative to its target components and also opens up the possibility of monitoring membrane fouling and cleaning, the degradation of organic substances, and the inactivation of pathogenic microorganisms [7,8].

Electrically conductive membranes for filtration are produced by the formation of conductive layers on ceramic or polymeric supports using metals, nanoporous carbon, carbon nanotubes (CNT), graphene, and conductive polymers [9,10]. The surface of the selective layer can acquire a charge by the injection or withdrawal of electrons due to an externally applied surface potential (electronic charge) and due to the dissociation of surface functional groups or the adsorption of charged species from solution (chemical charge). Surfaces with the simultaneous presence of electronic and chemical charges are known as amphifunctionally electrified solid–liquid interfaces [11,12]. For filtration applications, the membrane surface should be electrochemically stable in the range of applied potentials, i.e., no electrochemical reactions leading to membrane degradation and/or the production of undesirable species should occur during the filtration process [13].

The possibility to control ionic selectivity with an electric field was first demonstrated for track-etched membranes with gold-coated pores [14,15]. Experiments on the diffusion of ionic species through membranes have shown that their fluxes can be controlled by changing the applied potential [16,17]. The principal possibility of increasing the ionic conductivity of membranes by an order of magnitude due to the external potential was also demonstrated [18,19]. A new type of highly porous (60%) membrane based on alumina nanofibers with a diameter of about 10 nm covered by a conductive carbon coating was proposed in [20]. The possibility to control the ionic selectivity and conductivity of these membranes by changing the surface potential was demonstrated [21,22].

One of the first applications of electrically assisted membranes to nanofiltration was described in [23]. It was shown that a membrane based on nanotubes and conducting polymers can increase the rejection of monovalent ions from 50% to 80% without decreasing the permeability (14 L/m${}^{2}$ h bar). Similar results were obtained using membranes with a selective layer made of nanotubes and graphene oxide [24], although their permeability was much lower and decreased when increasing the applied voltage. It was shown in [25] that the rejection of dyes by an MXene/CNT nanofiltration membrane can be significantly improved by applying a cathode potential to the membrane surface. The selectivity control between monovalent and multivalent salt ions using a transmembrane electric field was demonstrated in [26]. The electric field created by the membrane surface was used to improve the efficiency of the microfiltration of silica and PS spheres, latex particles, phenols, and natural organic matter [27,28].

Mathematical models have been widely employed to understand and predict the complex hydrodynamic and physicochemical mechanisms of nanofiltration [29,30,31]. The first attempts to describe NF employed phenomenological equations derived from irreversible thermodynamics [32] considering the membrane as a black box. The pore flow model based on the Poisson–Nernst–Planck (PNP) equations coupled with the Navier–Stokes (NS) equations was developed and validated in [33,34,35]. This is also known as a space–charge (SC) model since it takes into account the variation in the electrical potential, ion concentrations, and pressure in the radial and axial directions of the pore. Later on, the Donnan model of the exclusion of ions was combined with the steric exclusion model (DSPM model) [36] and dielectric exclusion model (DSPM–DE model) [37,38]. This resulted in more realistic (reduced) values of surface charge density that were extracted by fitting the experimental data. The concentration polarization effect in cross-flow nanofiltration was theoretically investigated in [39]. A comparison between ion transport mechanisms in NF membranes with a constant surface potential and a constant surface charge was performed in [40,41] using two-dimensional NS–PMP equations. The space–charge (SC) model was extended to the case of electrically conductive nanopores in [42]. It was shown that the diffusion of ions through a membrane induces polarization charges on the nanopore’s surface, leading to the enhancement of the membrane potential at zero current. The polarization of the membrane surface can also result in a non–linear dependence of the streaming potential on the applied pressure [43]. The interaction between an electronic charge and a pH-dependent chemical charge and their impact on the membrane potential were theoretically analyzed in [44]. 1D and 2D models describing the transport of ions under concentration and electrical potential gradients in conductive membranes were proposed and validated against experimental data in [45,46].

In this work, we develop a mathematical model of nanofiltration with electrically assisted membranes, taking into account the electrical and chemical charging mechanisms. The model predictions are compared with recently obtained experimental data for composite membranes prepared from carbon nanotubes and conducting polymers or MXene nanosheets [23,25]. A parametric study of the dependence of rejection on model parameters is also performed.

## 2. Model Description

### 2.1. Problem Statement

**Figure 1:**
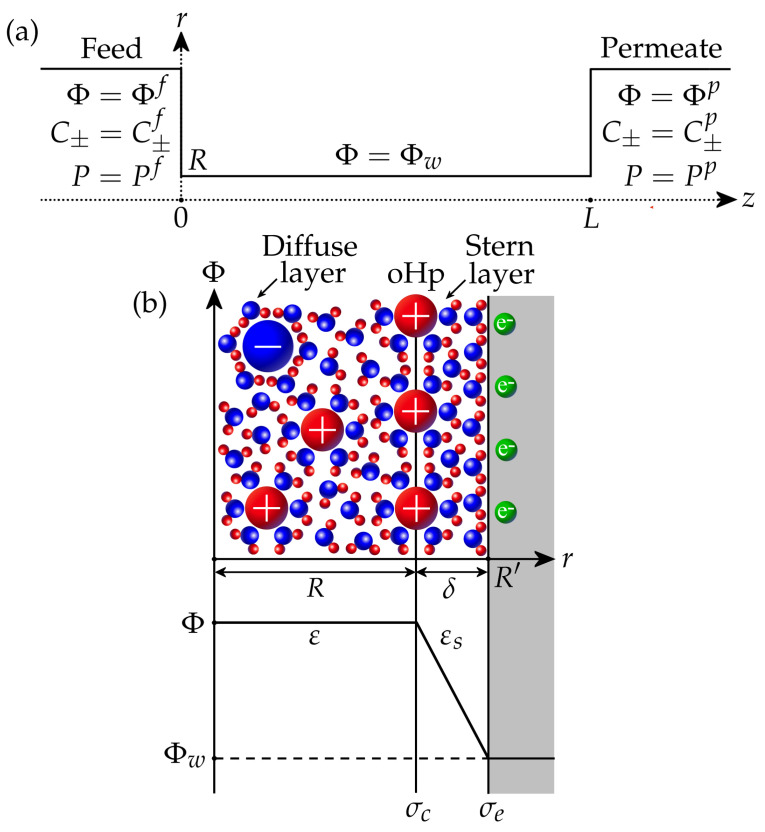
(**a**) A nanopore connecting feed and permeate sides. (**b**) The scheme of electric double layer in the nanopore.

We consider a binary aqueous electrolyte with cations and anions having charge numbers $z_{+}$ and $z_{-}$, respectively. The membrane is modelled as an array of straight nanopores with a size 2R and length *L*. If cylindrical nanopores are considered, *R* is the pore radius, while for plane nanopores, *R* is the half–width. Thus, it is sufficient to calculate the ion transport inside a single pore connecting feed (*f*) and permeate (*p*) sides, where the potential Φ, ion concentrations $C_{\pm }$, and pressures *P* are specified (Figure 1a). For the filtration problem, the pressure in the permeate is assumed to be zero without loss of generality, so the applied pressure difference is $\Delta P=P^{f}-P^{p}=P^{f}$. Similarly, the feed potential is fixed at $\Phi^{f}=0$, so $\Delta \Phi =\Phi^{p}-\Phi^{f}=\Phi^{p}$ is the filtration potential. The feed salt concentration is set as $C^{f}$, from which the feed ion concentrations are determined by

(1) $$C_{\pm }^{f}=C^{f}\left|z_{\mp }\right|.$$

The permeate ion concentrations and potential have to be determined. The electroneutrality condition holds in the feed and permeate sides as

$$\begin{array}{c} z_{+}C_{+}^{p,f}+z_{-}C_{-}^{p,f}=0. \end{array}$$

It is assumed that the walls of the membrane pores have electronic conductivity (Figure 1b). The *electronic* surface charge $\sigma_{e}$ is associated with an excess or deficiency of electrons and can be controlled by applying a given potential $\Phi_{w}$ to the surface via an external power source. The interaction of the electrolyte with the surface results in the formation of a *chemical* surface charge $\sigma_{c}$ due to the ion adsorption and/or dissociation of surface functional groups (for example, protonation/deprotonation). This may depend on the concentration of ions, the pH of the solution, the potential inside the pore, etc. In this work, we assume that the chemical charge is constant. The pore interior is divided into the Stern layer and the diffuse layer (Figure 1b). The Stern layer of thickness δ (typically 0.2–0.5 nm) is adjacent to the wall and contains only water molecules oriented by the strong electric field of the conducting wall [11,47]. As a result, its relative dielectric permittivity $\varepsilon_{s}$ decreases in comparison with the permittivity ε of the diffuse layer, which contains electrolyte ions and water molecules. It is assumed that the effective pore size 2R corresponds to the size of the diffuse layer, where mobile ions are located, while the total pore size is $2R^{\prime }=2R+2\delta$. The interface between the Stern and diffuse layers is known as the outer Helmholtz plane (oHp). It is assumed that the chemical surface charge $\sigma_{c}$ resides at this plane and is separated from the electronic charge $\sigma_{e}$ by the Stern layer.

The solution of the Poisson equation for electrical potential in the Stern layer provides the following expression for the surface charge density σ at the oHp [22,45]:

(2) $$\begin{array}{c} \sigma =\sigma_{e}+\sigma_{c}=c_{s}\left(\Phi_{w}-\Phi \right)+\sigma_{c}. \end{array}$$

Here, $c_{s}$ is the Stern layer capacitance. For a plane pore, it is given by $c_{s}=\varepsilon_{s}\varepsilon_{0}/\delta$, while the expression for a cylindrical pore is $c_{s}=\varepsilon_{s}\varepsilon_{0}{\left(R\ln\left(1+\delta /R\right)\right)}^{-1}$, see [45]. The quantity σ can be interpreted as the local surface charge density, which should be placed to the oHp to balance the diffuse layer charge.

In what follows, we assume that the pore size is comparable or smaller than the Debye length,

$$\begin{array}{c} \lambda =\sqrt{\frac{\varepsilon \varepsilon_{0}R_{g}T}{2C^{\prime }F^{2}}}, \end{array}$$

where $R_{g}$ is the ideal gas constant, *T* is the temperature, *F* is the Faraday constant, and $C^{\prime }$ is the reference salt concentration ($C^{f}<C^{\prime }<C^{p}$). Then, the electrical potential Φ, ion concentrations $C_{\pm }$, and pressure *P* can be considered homogeneous (constant) in any cross-section of the pore. Therefore, these quantities only depend on the coordinate *z* along the pore; see Figure 1a.

### 2.2. Governing Equations

The ion transport in the membrane pores is described by the extended Nernst–Planck equation [48]

(3) $$\begin{array}{c} J_{\pm }=K_{\pm }C_{\pm }J_{V}-K_{\pm }\varepsilon D_{\pm }\left(\frac{dC_{\pm }}{dz}+z_{\pm }C_{\pm }\frac{F}{R_{g}T}\frac{d\Phi }{dz}\right). \end{array}$$

Here, $J_{\pm }$ are transmembrane ion fluxes (mol/m${}^{2}$ s) and $J_{V}$ is the solvent flux or velocity (m${}^{3}$/m${}^{2}$ s). They are defined per unit geometric membrane area. To obtain the pore-based fluxes, the above quantities should be divided by the membrane transport reduction factor ε, which is the ratio of membrane porosity to pore tortuosity. Furthermore, $K_{\pm }$ are the friction factors, which incorporate the effects of friction between ions and water and between ions and the membrane matrix [48]. The value $K_{\pm }=1$ corresponds to no friction, while in the limiting case of $K_{\pm }=0$, the friction is such that the ion fluxes are absent. Finally, $D_{\pm }$ are ion diffusion coefficients in a free solution.

For a dead-end filtration process, the ion fluxes are related to solvent (water) flux as

(4) $$\begin{array}{c} J_{\pm }=C_{\pm }^{p}J_{V}. \end{array}$$

Relationship (4) is also valid for cross-flow filtration if the flows along the membrane can be neglected. In particular, this is the case for low water recovery, which corresponds to the situation when the ratio of permeate flux to the feed flux is low [49].

The electroneutrality condition inside the membrane is written as

(5) $$\begin{array}{c} z_{+}C_{+}+z_{-}C_{-}+X=0, \end{array}$$

where *X* is the membrane volume charge density, which is obtained by relating the total membrane surface charge to the total pore volume. In the case of cylindrical pores with a radius *R*, this relationship is given by X=2σ/RF. Taking into account Expression (2), the membrane volume charge density can be represented as

(6) $$\begin{array}{c} X=C_{s}\left(\Phi_{w}-\Phi \right)+X_{c}. \end{array}$$

Here,

(7) $$C_{s}=\frac{2c_{s}}{RF}$$

is the volume Stern layer capacitance (mol/m${}^{3}$V) and

(8) $$X_{c}=\frac{2\sigma_{c}}{RF}$$

is the volume chemical charge density. In what follows, we will also use the volume charge density and diffuse layer potential (at the oHp) averaged along the membrane pore

$$\bar{X}=\frac{1}{L}\int_{0}^{L}X\;dz,\;\bar{\Phi }=\frac{1}{L}\int_{0}^{L}\Phi \;dz.$$

The relationship between the solvent flux and gradients of pressure and potential is given by

(9) $$\begin{array}{c} J_{V}=AL\left(-\frac{dP}{dz}+FX\frac{d\Phi }{dz}\right). \end{array}$$

In this equation, *A* is the membrane permeability (L/m${}^{2}$ h bar). The first term describes the pressure-driven flow, while the second term corresponds to the electro-osmotic flow of solvent induced by the motion of ions in the electric field. For a membrane with parallel cylindrical pores, the permeability is calculated as

(10) $$\begin{array}{c} A=\frac{\varepsilon R^{2}}{8\mu L}. \end{array}$$

Let us express the ion concentration gradients from (3), resulting in

(11) $$\begin{array}{c} \frac{dC_{+}}{dz}=\frac{C_{+}K_{+}J_{V}-J_{+}}{\varepsilon K_{+}D_{+}}-\frac{F}{R_{g}T}z_{+}C_{+}\frac{d\Phi }{dz}, \end{array}$$

(12) $$\begin{array}{c} \frac{dC_{-}}{dz}=\frac{C_{-}K_{-}J_{V}-J_{-}}{\varepsilon K_{-}D_{-}}-\frac{F}{R_{g}T}z_{-}C_{-}\frac{d\Phi }{dz}. \end{array}$$

Now, we differentiate the electroneutrality condition (5) with respect to *z* and substitute (11) and (12) as well as the consequence $dX/dz=-C_{s}\;d\Phi /dz$ from (6) into the resulting expression. This leads to

(13) $$\begin{array}{c} \frac{d\Phi }{dz}=\frac{R_{g}T}{F}\left(z_{+}\frac{C_{+}K_{+}J_{V}-J_{+}}{\varepsilon K_{+}D_{+}}+z_{-}\frac{C_{-}K_{-}J_{V}-J_{-}}{\varepsilon K_{-}D_{-}}\right){\left(z_{+}^{2}C_{+}+z_{-}^{2}C_{-}+\frac{C_{s}R_{g}T}{F}\right)}^{-1}. \end{array}$$

Finally, the pressure gradient can be expressed from (9) as:

(14) $$\begin{array}{c} \frac{dP}{dz}=-\frac{J_{V}}{AL}+FX\frac{d\Phi }{dz}. \end{array}$$

### 2.3. Boundary Conditions

The boundary conditions at the solution–membrane interface (z=0) describe the potential and ion concentration jumps resulting from the Donnan equilibrium and other ion-partitioning mechanisms, as well as the osmotic pressure jump:

(15) $$\begin{array}{ccc} & & \Phi \left(0\right)=\Phi_{0}, \end{array}$$

(16) $$\begin{array}{ccc} & & C_{\pm }\left(0\right)=C_{\pm }^{f}\phi_{\pm }\exp\left(-z_{\pm }\left(\Phi \left(0\right)-\Phi^{f}\right)\;F/R_{g}T\right), \end{array}$$

(17) $$\begin{array}{ccc} & & P\left(0\right)=P^{f}+R_{g}T\left(C_{+}\left(0\right)-C_{+}^{f}+C_{-}\left(0\right)-C_{-}^{f}\right). \end{array}$$

Here, $\phi_{\pm }$ are the partition coefficients that include all effects (steric exclusion, dielectric exclusion, etc.), except the Donnan equilibrium. For a cylindrical pore with radius *R*, the steric partition coefficients are calculated as $\phi_{\pm }={\left(1-r_{\pm }/R\right)}^{2}$, where $r_{\pm }$ are the ion radii. The electrical potential $\Phi_{0}$ at z=0 can be determined from the electroneutrality condition (5), taking into account (6),

(18) $$\begin{array}{c} z_{+}C_{+}\left(0\right)+z_{-}C_{-}\left(0\right)+C_{s}\left(\Phi_{w}-\Phi_{0}\right)+X_{c}=0, \end{array}$$

where $C_{\pm }\left(0\right)$ are given by (16).

At the permeate side, the corresponding boundary conditions are written as

(19) $$\begin{array}{ccc} & & \Phi \left(L\right)=\Phi_{L}, \end{array}$$

(20) $$\begin{array}{ccc} & & C_{\pm }\left(L\right)=C_{\pm }^{p}\phi_{\pm }\exp\left(-z_{\pm }\left(\Phi \left(L\right)-\Phi^{p}\right)\;F/R_{g}T\right), \end{array}$$

(21) $$\begin{array}{ccc} & & P\left(L\right)=P^{p}+R_{g}T\left(C_{+}\left(L\right)-C_{+}^{p}+C_{-}\left(L\right)-C_{-}^{p}\right). \end{array}$$

### 2.4. Concentration Polarization

The effect of concentration polarization leads to an increase in salt concentration just near the membrane surface in comparison with the feed concentration [50,51]. Let us introduce the total salt concentration $C=C_{\pm }/\left|z_{\mp }\right|$ and the total salt flux $J=J_{\pm }/\left|z_{\mp }\right|$. Then, the transport of salt can be described by the equation [52]

(22) $$\begin{array}{c} J=CJ_{V}-D\frac{dC}{dz}. \end{array}$$

Here, *D* is the harmonic average of the ion diffusion coefficients

$$\begin{array}{c} D=\frac{|z_{+}\left|+\right|z_{-}|}{|z_{-}|/D_{+}+\left|z_{+}\right|/D_{-}}. \end{array}$$

The solution of (22) in the concentration polarization layer of thickness $L_{d}$ with the boundary condition $C\left(-L_{d}\right)=C^{f}$ provides the following expression for salt concentration $C\left(0\right)=C^{m}$ just near the membrane surface:

(23) $$\begin{array}{c} C^{m}=\left(C^{f}-\frac{J}{J_{V}}\right)\exp\left(\frac{J_{V}}{k_{d}}\right)+\frac{J}{J_{V}}=\left(C^{f}-C^{p}\right)\exp\left(\frac{J_{V}}{k_{d}}\right)+C^{p}. \end{array}$$

Here, we have employed the relationship $J=C^{p}J_{V}$, which follows from (4). In the above expression, $k_{d}=D/L_{d}$ is the mass transfer coefficient with its dimension in m/s or L/m${}^{2}$ h = LMH. When the concentration polarization is taken into account, the value of $C^{m}$ should be used instead of $C^{f}$ in the boundary conditions at the feed side; see (1) and (15)–(17).

For a cross-flow filtration setup, where the solution flows along the membrane inside a gap of height *H* and length $L_{m}$ with a velocity *u*, the mass transfer coefficient is determined by [53,54]

(24) $$\begin{array}{c} k_{d}=Sh\frac{D}{H}. \end{array}$$

Here, Sh is the Sherwood number, which follows the correlation

(25) $$\begin{array}{c} Sh=1.85\;{\left(R\;Pe\right)}^{1/3}, \end{array}$$

where $R=H/L_{m}$ and Pe=uH/D is the Peclet number.

### 2.5. Numerical Implementation

The algorithm for solving the model equations can be summarized as follows:

Step 1. Set the values of the potential, ion concentrations, and pressure in the feed reservoir, as well as the zero permeate pressure.

Step 2. Set the initial approximations for the permeate potential $\Phi^{p}$, cation concentration $C_{+}^{p}$, and solvent flux $J_{V}$.

Step 3. Calculate the salt concentration just near the membrane surface according to (23).

Step 4. Determine the potential $\Phi_{0}$ by the numerical solution of Equation (18).

Step 5. Set the boundary conditions at z=0 according to Equations (15)–(17).

Step 6. Numerically integrate Equations (11)–(14) from z=0 to z=L.

Step 7. Determine the next approximations for $\Phi^{p}$, $C_{+}^{p}$, and $J_{V}$ from the condition that the left-hand sides of the three boundary conditions in (20) and (21) must be equal to the right-hand sides.

Step 8. Iterate Steps 3–7 until the convergence for the permeate potential, cation concentration, and solvent flux is achieved with the desired accuracy.

To integrate the model equations, the Runge–Kutta–Merson method of the 5th order of accuracy with a variable step was used. The fitting of model parameters to the experimental data was performed by minimizing the sum of squared errors using the golden section method (for a single-parameter fit) and the Nelder–Mead simplex method (for a multiple-parameter fit).

## 3. Results and Discussion

### 3.1. Physical Parameters

We start with a parametric study, which shows the influence of different parameters on the membrane charge, salt rejection, and transmembrane flux. The set of physical parameters employed in this study is presented in Table 1. In the calculations below, a number of parameters are varied, while the rest are fixed to the values given in the table. These values are typical for the low-pressure nanofiltration of aqueous salt solutions [23,24,25,26]. In particular, the diffusion coefficients and ion radii correspond to a NaCl aqueous solution [55]. The chosen membrane thickness corresponds to the thickness of the selective layer, while the pore size is actually the size of the diffuse layer where the mobile ions are present; see Figure 1.

### 3.2. The Influence of Electronic Charge

The electronic charge of a membrane is controlled by the surface potential $\Phi_{w}$. It is proportional to the difference between the potentials at the membrane surface and the outer Helmholtz plane, and the Stern layer capacitance plays the role of the proportionality coefficient; see Formulas (2) and (6).

**Figure 2:**
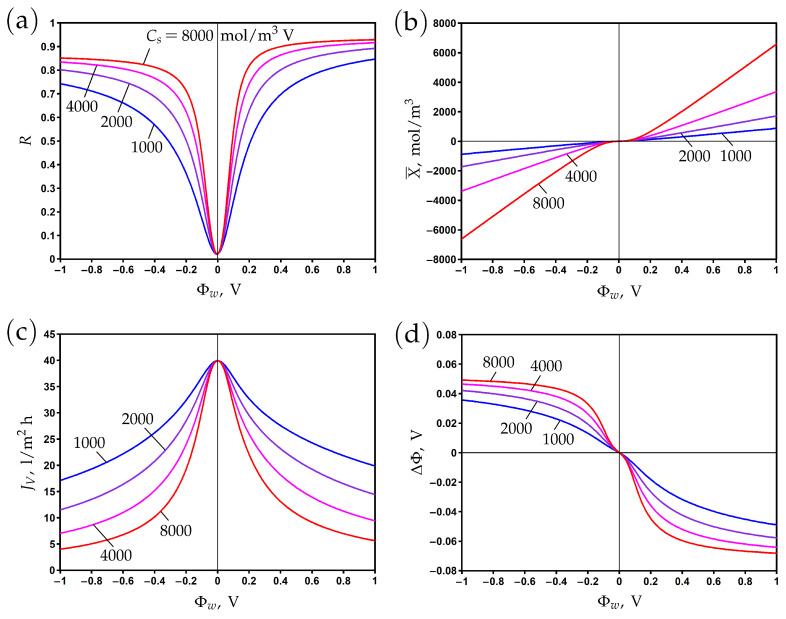
The dependence of rejection (**a**), membrane volume charge density (**b**), transmembrane flux (**c**), and membrane potential (**d**) on the potential applied to the membrane surface for different values of Stern layer volume capacitance.

The variation of rejection with surface potential is presented in Figure 2a for different values of the Stern layer volume capacitance. The value $C_{s}=2000$ mol/m${}^{3}$ V corresponds to the Stern layer capacitance $c_{s}\;=\;0.096$ F/m${}^{2}$ and the relative dielectric permittivity $\varepsilon_{s}$ = 3.4 assuming that the Stern layer thickness is δ=0.5 nm; see Formula (7). These are typical values that characterize the effect of a reduction in the dielectric permittivity due to the orientation of water molecules in the strong electric field near the electrically conductive pore surface [11,12].

In the absence of a chemical charge ($X_{c}=0$), the rejection reaches its minimum at $\Phi_{w}=0$, where the electronic charge vanishes. Variations of the surface potential in the positive or negative direction lead to corresponding changes in the electronic charge (Figure 2b). The rejection increases accordingly due to the Donnan exclusion of co-ions. When the Stern layer capacitance becomes larger, an increase in rejection is observed due to the increase in electronic charge. Note that the correlation between $\bar{X}$ and $\Phi_{w}$ is linear according to Formula (6), but at low potentials, the averaged second term in the right-hand side of this formula provides a non-linear contribution.

The asymmetry of rejection with respect to the origin is explained by the difference of ion diffusion coefficients. In particular, the rejection is higher (lower) for positive (negative) potentials due to a lower (higher) sodium (chloride) co-ion diffusion coefficient. The steric effect leads to the opposite trend due to the smaller (larger) size of sodium (chloride) ions, but its influence on rejection is much smaller than that of diffusion.

The transmembrane flux reaches its maximum when the membrane is uncharged and decreases when the absolute value of the surface potential increases (Figure 2c). It results from an increase in the osmotic pressure difference due to a reduction in permeate concentration, which in turn reduces the total pressure difference when the hydrodynamic pressure difference is fixed. The second reason for the decrease in flux with the increase in electronic charge is the electro-osmotic flow directed against the applied pressure gradient; see the second term in the right-hand side of Equation (9).

The difference between potentials on the permeate and feed sides (membrane or filtration potential) is determined by the counter-ion charge. Thus, it is positive (negative) for negative (positive) surface potentials where the cation (anion) is the counter-ion; see Figure 2d.

### 3.3. The Influence of Pressure Difference

**Figure 3:**
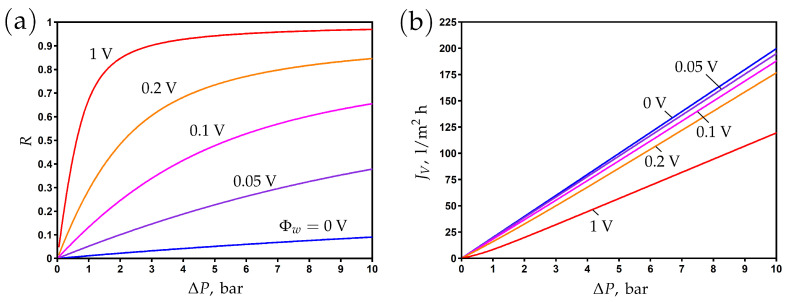
The dependence of rejection (**a**) and transmembrane flux (**b**) on the applied pressure difference for different values of surface potential.

The impact of pressure difference on rejection is shown in Figure 3a for different values of the surface potential. The rejection increases with an increase in applied pressure and surface potential. It is clear that the enhancement of the electronic charge by varying the surface potential can significantly increase the salt rejection. In other words, the same rejection can be achieved at much lower pressures due to the enhancement of the membrane charge.

The transmembrane flux increases linearly with the applied pressure; see Figure 3b. The application of this potential leads to a decrease in flux due to an increase in osmotic pressure and the development of electro-osmotic flow in the opposite direction of the pressure-driven flow.

### 3.4. The Influence of Chemical Charge

**Figure 4:**
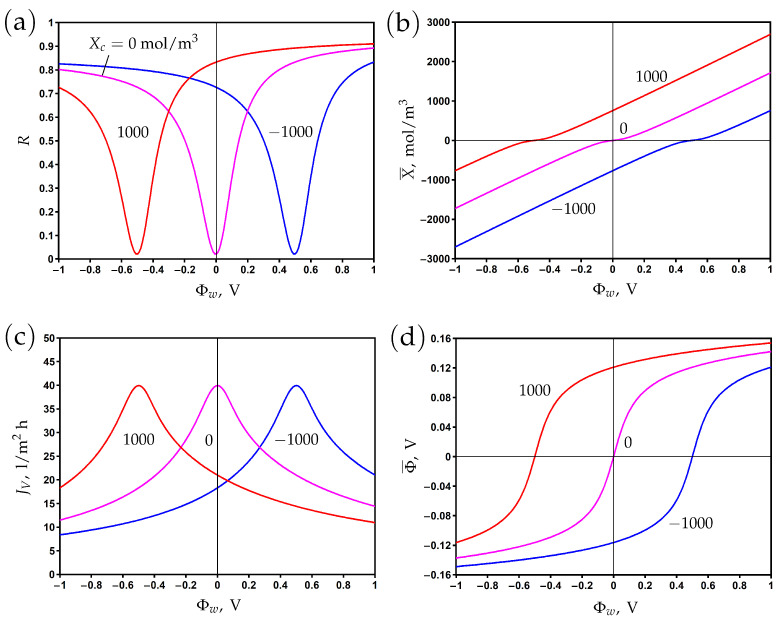
The dependence of rejection (**a**), membrane volume charge density (**b**), transmembrane flux (**c**), and averaged diffuse layer potential (**d**) on the potential applied to the membrane surface for different values of chemical charge density.

If the chemical charge is absent, then the membrane charge vanishes at zero surface potential and becomes positive or negative when the potential is positive or negative, respectively; see Figure 2b. The rejection curve reaches its minimum at $\Phi_{w}=0$, which is the potential of zero charge (PZC). For a positive (negative) chemical charge, this shifts to negative (positive) potential values (Figure 4b). This is due to the screening of electronic charges by the chemical charge. The rejection curve shifts accordingly, reaching its minimum at PZC (Figure 4a). At the same time, the transmembrane flux reaches its maximum at this point and decreases for both positive and negative directions of the surface potential; see Figure 4c. The variations in the diffuse layer potential are shown in Figure 4d, which shows that it shifts to positive (negative) values for positive (negative) chemical charges. The difference between the surface potential and diffuse layer potential corresponds to the potential decrease in the Stern layer; see also Figure 1b.

### 3.5. The Influence of Other Factors

The membrane transport reduction factor ε is determined by the ratio of membrane porosity to the pore tortuosity. The ion fluxes due to diffusion and electro-migration are proportional to this factor according to Equation (3). At the same time, the membrane permeability *A* and, consequently, the solvent flux $J_{V}$ are usually proportional to this factor; see (9) and (10). Thus, it follows from (3) and (4) that the variation of ε does not affect salt rejection.

Considering the influence of the ion-membrane friction factors $K_{\pm }$, we note that the ion fluxes are proportional to these factors. Therefore, their decrease results in a decrease in permeate ion concentrations according to Equation (4), as well as in a corresponding increase in salt rejection.

### 3.6. Comparison with Experimental Results: PANi–PSS/CNT Membranes

In this section, we apply the developed model to describe experimental data on the nanofiltration of aqueous salt solutions with the help of an electrically assisted membrane [23]. The membrane was prepared by the vacuum filtration of carbon nanotubes on a polyvinylidene fluoride (PVDF) substrate. After that, the polyaniline (PANi)/ polystyrenesulfonate (PSS) was polymerized into the CNT layer and in situ cross-linked with glutaraldehyde (GA) under acidic conditions.

The characteristics of the PANi–PSS/CNT membrane and the parameters of the filtration experiments are presented in Table 2. The two-electrode scheme was used to apply the potential to the membrane (working electrode), and a titanium mesh (counter electrode) was placed near the membrane. The potential difference between the counter and working electrodes was varied from 0 to 2.5 V. A separate study showed that under these conditions, the potential of the membrane with respect to the reference 4.2 M Ag/AgCl electrode varied from 0.05 to −1.28 V [23]. This potential range is used in the modelling study.

A cross-flow filtration cell was used in the experiments. The gap thickness above the membrane was H=1 mm, and the gap length was taken to be equal to the membrane diameter $L_{m}=32$ mm. The transmembrane velocity at a pressure difference of 2 bar was 8 μm/s.

The cross-flow velocity varied in the range of 0.03–0.1 m/s, and here, we use the value u=0.042 m/s to estimate the impact of concentration polarization. For an aqueous NaCl solution with an average diffusion coefficient $D=1.61\cdot 10^{-9}$ m${}^{2}$/s, Formulas (24) and (25) provide the mass transfer coefficient $k_{d}=100$ LMH.

**Figure 5:**
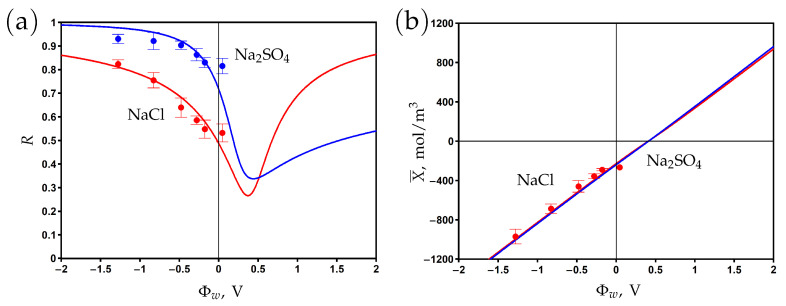
The dependence of rejection on the potential applied to the membrane surface for two aqueous salt solutions (**a**). Variations in membrane volume charge density with the surface potential (**b**). Experimental data (dots) [23] and model calculations (solid curves).

The surface of the PANi–PSS/CNT membrane is negatively charged due to the presence of sulfonic groups SO${}_{3}^{-}$ in the structure of PSS. The chemical charge density is estimated as −11.9 mC/m${}^{2}$ by measuring the ζ potential in 5 mM NaCl aqueous solution and using the Gouy–Chapmann equation [23]. The corresponding volume chemical charge density determined from Formula (8) is $X_{c}=-250$ mol/m${}^{3}$. The electronic charge density at different applied potentials was measured from the dependence of the charging current on time [23]. The experimental data on the dependence of the membrane charge are shown in Figure 5b. One can see that this dependence is linear, which agrees with Formula (6). The Stern layer volume capacitance of $C_{s}=617$ mol/m${}^{3}$ V is determined by fitting the experimental data with the help of the developed model. The modelling curve predicts the potential of zero charge at $\Phi_{w}^{pzc}=+0.4$ V. At this point, the negative chemical charge is screened by the positive electronic charge. The membrane is positively (negatively) charged at potentials lower (higher) than $\Phi_{w}^{pzc}$. The calculations show that the averaged diffuse layer potential varies from −62 to 33 mV for a sodium sulfate electrolyte and from −80 to 74 mV for a sodium chloride electrolyte when the surface potential changed from −1 to 1 V.

The experimental rejection data and theoretical curves are presented in Figure 5a for aqueous sodium chloride and sodium sulfate solutions. The curves are obtained by fitting the experimental data with two fitting parameters: the friction factor $K_{\pm }$ and the charge reduction factor ξ. The volume charge density *X* is multiplied by ξ in the model equations to describe the ’effective’ charge, which results in the observed rejection. The decrease in charge density might be explained by the complexation of sulfonic groups with Na${}^{+}$ ions as well as counter-ion adsorption on the (electrically) charged surface [14,15,16,17,49,57]. These effects are not explicitly treated in the present work since a separate study is required to determine the complexation/adsorption equilibrium parameters.

The fitted rejection curves demonstrate good agreement with the experimental data. The minimum rejection is observed near the PZC, while the rejection increases in both the negative and positive directions with respect to this point. The rejection is higher for sodium sulfate than for sodium chloride at potentials that are negative relative to the PZC due to the higher charge number of counter-ions (SO${}_{4}^{2-}$ vs Cl${}^{-}$). The opposite situation is observed at potentials that are positive relative to the PZC since the concentration of Na${}^{+}$ co-ions is two times higher for sodium sulfate than for sodium chloride at the same feed concentration, $C^{f}$; see (1).

**Figure 6:**
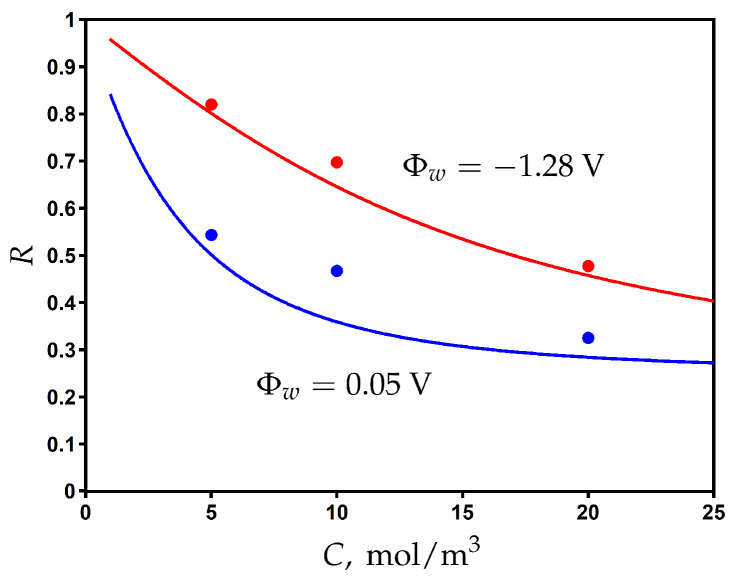
The variation of rejection with the feed concentration of NaCl for different values of potential applied to the membrane. Experimental data (dots) [23] and model calculations (solid curves).

Using the values obtained for fitting parameters, we plot the rejection as a function of feed concentration for different values of applied surface potential in Figure 6. The rejection decreases when increasing the feed concentration, but it can be significantly improved by electrical assistance when the applied surface potential is switched from 0.05 V to −1.28 V. The comparison with the experimental data shows a good agreement and provides additional validation of the proposed model.

### 3.7. Comparison with Experimental Results: MXene/CNT Membranes

Let us now consider the application of our proposed model to the nanofiltration membrane prepared from carbon nanotubes intercalated between MXene nanosheets [25]. The nanosheets were obtained by etching the Al of Ti${}_{3}$AlC${}_{2}$ using lithium fluoride (LiF) and hydrochloric acid (HCl). The selective layer was produced by the vacuum filtration of the uniform MXene/CNT dispersion on a hydrophilic PVDF membrane. The CNT intercalation prevented the MXene nanosheets from restacking and enriched the nanochannels for water transport.

The characteristics of the MXene/CNT membrane and the parameters of the filtration experiments are presented in Table 3. Aqueous solutions of methyl orange (MO) and prange G (OG) with concentrations of 20 mg/L were used in the cross-flow filtration experiments. A two-electrode scheme was employed to apply a voltage to the membrane. For each potential difference between the working electrode (membrane) and counter electrode (titanium mesh), the potential of the membrane surface with respect to the 4.2 M Ag/AgCl reference electrode was measured in a separate study. The range of the measured potentials is indicated in Table 3, and it is used in the present modelling study.

The surface of the carbon nanotubes and MXene nanosheets is terminated by oxygen-containing groups (−O−, −OH), which implies that the membrane surface has a negative chemical charge. Measurements of electronic charge were not performed in [25], so the value of the Stern layer volume capacitance was taken as close to that of the PANi–PSS/CNT membrane; see Table 2 and Table 3.

**Figure 7:**
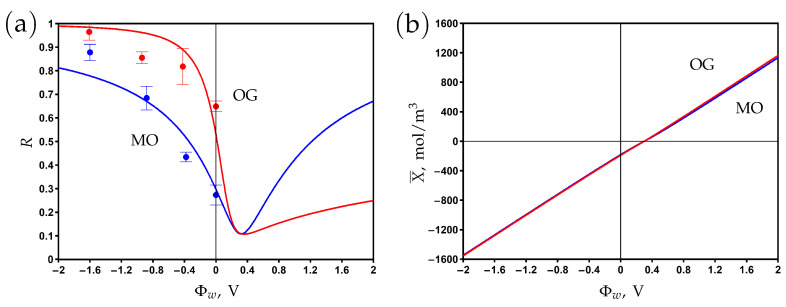
The dependence of rejection on the potential applied to the membrane surface for methyl orange (MO) and orange G (OG) aqueous solutions (**a**). The variation of membrane volume charge density with the surface potential (**b**). Experimental data (dots) [25] and model calculations (solid curves).

The dependence of the rejection and volume charge density on the surface potential is shown in Figure 7a and Figure 7b, respectively. The experimental data are fitted by the model using the friction factor and charge reduction factor as the fitting parameters. The rejection reaches its minimum at the potential of zero charge $\Phi_{w}^{pzc}=+0.3$ V, where the negative chemical charge is compensated by the positive electronic charge. The rejection increases when the potential varies in negative and positive directions with respect to the PZC. The asymmetry of the curves can be explained by the difference between dye anions and sodium cation diffusion coefficients and charge numbers. The rejection of OG with a charge number of −2 is higher than that of MO with a charge number of −1 at negative potentials. The significant difference between the rejection of OG at negative and positive potentials with respect to the PZC is explained by the difference in the charge and concentration of co-ions (the OG anion for $\Phi_{w}<\Phi_{w}^{pzc}$ and the sodium cation for $\Phi_{w}>\Phi_{w}^{pzc}$).

**Table 3:** The characteristics of MXene/CNT membrane, parameters of filtration experiments [25], ion properties [58,59,60], and model parameters.

Parameter	Dimension	Value
Membrane properties		
Average pore size 2R	nm	2
Thickness *L*	μm	0.502
Permeability *A* for MO	L/m${}^{2}$ h bar	27
Permeability *A* for OG	L/m${}^{2}$ h bar	25
Porosity ε	−	0.2
Parameters of filtration experiments		
Temperature *T*	K	298.15
Pressure difference ΔP	bar	1
MO feed concentration $C^{f}$	mol/m${}^{3}$	0.0611
OG feed concentration $C^{f}$	mol/m${}^{3}$	0.0884
Surface potential $\Phi_{w}$	V	−1.6...0
Ion properties		
Na${}^{+}$ radius	nm	0.095
Na${}^{+}$ diffusion coefficient	$10^{-9}$ m${}^{2}$/s	1.33
MO anion radius	nm	0.420
OG anion radius	nm	0.550
MO anion charge number	−	−1
OG anion charge number	−	−2
MO anion diffusion coefficient	$10^{-9}$ m${}^{2}$/s	0.91
OG anion diffusion coefficient	$10^{-9}$ m${}^{2}$/s	0.70
Model parameters		
Stern layer thickness δ	nm	0.5
Stern layer volume capacitance $C_{s}$	mol/m${}^{3}$ V	700
Volume chemical charge density $X_{c}$	mol/m${}^{3}$	−200
Friction factor $K_{\pm }$	−	0.18
Charge reduction factor ξ	−	8.03·10${}^{-4}$
Mass transfer coefficient $k_{d}$	L/m${}^{2}$ h	100

The low value of the fitted charge reduction factor (see Table 3) suggests that the effective membrane charge is much lower than that given by Formula (6). Similarly to the previously considered case of the PANi–PSS/CNT membrane, we believe that this occurs due to the complexation of surface groups with Na${}^{+}$ ions and/or their adsorption on the electrically charged surface at negative applied potentials.

## 4. Conclusions

In this work, we have developed a mathematical model that describes the performance of electrically assisted nanofiltration membranes for binary aqueous electrolytes. The model is based on the Nernst–Planck equations for ion fluxes supplemented by the electroneutrality condition and the relationship between the solvent flux and pressure gradient. The surface charge of membrane pores is formed by the chemical charge that occurred due to the ion adsorption and dissociation of functional groups and by the electronic charge, which is controlled by the potential of the electrically conductive membrane surface. The exclusion of ions by Donnan and steric mechanisms is assumed, and the concentration polarization effect is taken into account.

The influence of model parameters on membrane performance is first investigated theoretically. When the chemical charge is absent, the salt rejection reaches its minimum at the potential of zero charge (PZC), corresponding to zero applied potential. The increase in the surface potential magnitude enhances the rejection significantly but lowers the transmembrane flux due to the increase in the osmotic pressure difference and the development of electro-osmotic flow in the opposite direction of the pressure-driven flow. The presence of a positive (negative) chemical charge shifts the PZC and the minimum rejection in the direction of negative (positive) potentials due to the screening of the electronic charge.

The proposed model is applied to the description of experimental data on the rejection of salts and anionic dyes by the PANi–PSS/CNT and MXene/CNT nanofiltration membranes. The experimentally measured surface charge data allow us to determine the Stern layer volume capacitance and chemical charge. The rejection data are fitted by the model using the ion-membrane friction factor and charge reduction factor as fitting parameters. It is shown that the effective membrane charge is much smaller than the measured/fitted charge, which could be explained by the complexation of surface groups and/or the adsorption of counter-ions on the electrically charged surface.

The obtained results provide new insights into the mechanisms of ion rejection by electrically conductive membranes and could be employed to describe electrically assisted nanofiltration membrane processes. 

## Figures and Tables

**Table 1:** The list of physical parameters and their values employed in the present study.

Parameter	Dimension	Value
Temperature *T*	K	298.15
Pore size 2R	nm	2
Stern layer thickness δ	nm	0.5
Membrane thickness *L*	μm	2
Membrane permeability *A*	L/m${}^{2}$ h bar	20
Pressure difference ΔP	bar	2
Feed concentration $C^{f}$	mol/m${}^{3}$	10
Surface potential $\Phi_{w}$	V	0.1
Stern layer volume capacitance $C_{s}$	mol/m${}^{3}$ V	2000
Volume chemical charge density $X_{c}$	mol/m${}^{3}$	0
Cation charge number $z_{+}$	−	+1
Anion charge number $z_{-}$	−	−1
Cation radius $r_{+}$	nm	0.095
Anion radius $r_{-}$	nm	0.181
Diffusion coefficient $D_{+}$	$10^{-9}$ m${}^{2}$/s	1.33
Diffusion coefficient $D_{-}$	$10^{-9}$ m${}^{2}$/s	2.03
Friction factor $K_{\pm }$	−	1.0
Porosity reduction factor ε	−	0.2

**Table 2:** The characteristics of PANi–PSS/CNT membrane, parameters of filtration experiments [23,56], ion properties [55], and model parameters.

Parameter	Dimension	Value
Membrane properties		
Average pore size 2R	nm	2
Thickness *L*	μm	2.8
Permeability *A*	L/m${}^{2}$ h bar	14.5
Porosity ε	−	0.2
Parameters of filtration experiments		
Temperature *T*	K	298.15
Pressure difference ΔP	bar	2
Feed concentration $C^{f}$	mol/m${}^{3}$	5...20
Surface potential $\Phi_{w}$	V	−1.28...0.05
Ion properties		
Na${}^{+}$ radius	nm	0.095
Cl${}^{-}$ radius	nm	0.181
SO${}_{4}^{2-}$ radius	nm	0.290
Na${}^{+}$ diffusion coefficient	$10^{-9}$ m${}^{2}$/s	1.33
Cl${}^{-}$ diffusion coefficient	$10^{-9}$ m${}^{2}$/s	2.03
SO${}_{4}^{2-}$ diffusion coefficient	$10^{-9}$ m${}^{2}$/s	1.06
Model parameters		
Stern layer thickness δ	nm	0.5
Stern layer volume capacitance $C_{s}$	mol/m${}^{3}$ V	617
Volume chemical charge density $X_{c}$	mol/m${}^{3}$	−250
Friction factor $K_{\pm }$	−	0.16
Charge reduction factor ξ	−	0.079
Mass transfer coefficient $k_{d}$	L/m${}^{2}$ h	100

## Data Availability

Data is contained within the article.

## Nomenclature

*r*	Transversal coordinate, m
*z*	Longitudinal coordinate, m
*R*	Pore radius or half-width, m
*L*	Pore length/selective layer thickness, m
$z_{\pm }$	Cation/anion charge number
$r_{\pm }$	Cation/anion radius, m
$C_{\pm }$	Concentration of cations/anions, mol/m${}^{3}$
$J_{\pm }$	Cation/anion flux, mol/m${}^{2}$ s
$D_{\pm }$	Cation/anion diffusion coefficient, m${}^{2}$/s
$K_{\pm }$	Friction factor
$\phi_{\pm }$	Steric factor
*D*	Salt diffusion coefficient, m${}^{2}$/s
$J_{V}$	Solvent flux, m${}^{3}$/m${}^{2}$ s
Φ	Electrical potential, V
$\Phi_{w}$	Surface potential, V
*P*	Pressure, Pa
ΔP	Pressure difference, Pa
σ	Total surface charge density, C/m${}^{2}$
$\sigma_{e}$	Electronic surface charge density, C/m${}^{2}$
$\sigma_{c}$	Chemical surface charge density, C/m${}^{2}$
*X*	Total volume charge density, mol/m${}^{3}$
$X_{c}$	Volume chemical charge density, mol/m${}^{3}$
$c_{s}$	Stern layer capacitance, F/m${}^{2}$
$C_{s}$	Stern layer volume capacitance, mol/m${}^{3}$ V
δ	Stern layer thickness, m
$\varepsilon_{0}$	Dielectric constant, F/m
$\varepsilon_{s}$	Stern layer relative permittivity
ε	Membrane porosity
*T*	Temperature, K
$R_{g}$	Universal gas constant, J/kg·K
*F*	Faraday constant, C/mol
*A*	Membrane permittivity, m${}^{3}$/m${}^{2}$ h bar
μ	Solvent dynamic viscosity, Pa·s
$k_{d}$	Mass transfer coefficient, m${}^{3}$/m${}^{2}$ h
*u*	Cross-flow velocity, m/s
*H*	Gap height in a cross-flow cell, m
$L_{m}$	Gap length in a cross-flow cell, m
$L_{d}$	Concentration polarization layer thickness, m
*R*	Aspect ratio of the gap
Pe	Peclet number
Sh	Sherwood number
*Indices*
	*f*	feed
*p*	permeate
